# Hypothermia Improves Oral and Gastric Mucosal Microvascular Oxygenation during Hemorrhagic Shock in Dogs

**DOI:** 10.1155/2013/589606

**Published:** 2013-11-12

**Authors:** Christian Vollmer, Ingo Schwartges, Meike Swertz, Christopher Beck, Inge Bauer, Olaf Picker

**Affiliations:** Department of Anesthesiology, University Hospital Duesseldorf, Moorenstrasse 5, 40225 Duesseldorf, Germany

## Abstract

Hypothermia is known to improve tissue function in different organs during physiological and pathological conditions. The aim of this study was to evaluate the effects of hypothermia on oral and gastric mucosal microvascular oxygenation (**μ**HbO_2_) and perfusion (**μ**flow) under physiological and hemorrhagic conditions. Five dogs were repeatedly anesthetized. All animals underwent each experimental protocol (randomized cross-over design): hypothermia (34°C), hypothermia during hemorrhage, normothermia, and normothermia during hemorrhage. Microcirculatory and hemodynamic variables were recorded. Systemic (DO_2_) and oral mucosal (**μ**DO_2_) oxygen delivery were calculated. Hypothermia increased oral **μ**HbO_2_ with no effect on gastric **μ**HbO_2_. Hemorrhage reduced oral and gastric **μ**HbO_2_ during normothermia (−36 ± 4% and −27 ± 7%); however, this effect was attenuated during additional hypothermia (−15 ± 5% and −11 ± 5%). The improved **μ**HbO_2_ might be based on an attenuated reduction in **μ**flow during hemorrhage and additional hypothermia (−51 ± 21 aU) compared to hemorrhage and normothermia (−106 ± 19 aU). **μ**DO_2_ was accordingly attenuated under hypothermia during hemorrhage whereas DO_2_ did not change. Thus, in this study hypothermia alone improves oral **μ**HbO_2_ and attenuates the effects of hemorrhage on oral and gastric **μ**HbO_2_. This effect seems to be mediated by an increased **μ**DO_2_ on the basis of increased **μ**flow.

## 1. Introduction

The gastrointestinal tract is not only responsible for nutrient absorption but also functions as a metabolic and immunological system, forming an effective barrier against endotoxins and bacteria in the intestinal lumen. Maintenance of this mucosal barrier function by improving perfusion and oxygenation seems to be of vital importance [1–3].

However, during severe illness (e.g., septic or hypovolemic shock) blood flow is redistributed and splanchnic oxygenation is impaired early to preserve perfusion of more vital organs (i.e., heart and brain) [4, 5]. Insufficient microcirculatory oxygen supply impairs mucosal barrier function and has been shown to enable translocation of bacteria and bacterial toxins into portal venous and local lymphatic circulation [6] and to mediate an inflammatory response syndrome [7]. Therefore, adequate splanchnic perfusion and in particular oxygenation of the gastrointestinal mucosa are considered crucial for the prevention and therapy of critical illness [1, 8, 9]. Alterations of the oral microcirculation are an independent predictor of organ failure and associated with a high mortality [10, 11]. Thus, growing effort is made to develop strategies to improve splanchnic mucosal oxygenation and to avoid tissue hypoxia. Under these circumstances, especially during hypoxia, hypothermia is known to improve tissue function in a variety of tissues, for example, heart, brain, liver, and spinal cord [12–14] during trauma, anemia, neonatal asphyxia, respiratory failure, reduced inspiratory oxygen, and carbon monoxide intoxication [14, 15]. In contrast, hypothermia has also been shown to exert negative effects like a reduction of CO, cardiac arrhythmia, immunosuppression with an increased risk for infection, and an impaired coagulation cascade [14].

The impact of hypothermia on gastrointestinal circulation is controversially discussed. In a hemorrhagic shock model in rats hypothermia led to an increase in blood flow [16]. In contrast, in a study in piglets, blood flow decreased during hypothermia in separate layers of the intestinal wall [17]. It is yet unclear if the observed changes in blood flow have any impact on bacterial translocation from the gastrointestinal lumen. Hypothermia during hemorrhagic shock decreased bacterial translocation into the spleen, liver, and mesenteric lymph nodes [16], but also opposing results with increased bacterial translocation linked to hypothermia were published [18]. Another study did not observe any changes in bacterial translocation during hemorrhagic shock under hypothermia but hypothermia improved survival [19]. Since hemorrhage reduces gastric mucosal oxygenation, the effects of hypothermia on gastric mucosal oxygenation during hemorrhage are of particular importance. Despite intense effort to analyze the effects of hypothermia on splanchnic perfusion, there are no studies investigating the effect on splanchnic oxygenation.

The effect of hypothermia on splanchnic mucosal oxygenation is of clinical interest for mainly two reasons. Hypothermia is already widely used as a therapeutic approach in critical illness, for example, after resuscitation, and is currently discussed as a therapeutic approach with additional indications [20] without detailed knowledge about possible negative effects on splanchnic oxygenation. As hypothermia is protective in tissue hypoxia of miscellaneous tissues, it might have a positive impact on splanchnic oxygenation, especially on local hypoxia during hemorrhage.

The aim of our study was to evaluate the effects of hypothermia on splanchnic mucosal oxygenation and perfusion. We investigated two representative mucosal regions (oral and gastric) under physiological and hemorrhagic conditions.

## 2. Materials and Methods

### 2.1. Animals

The data were derived from repetitive experiments on five dogs (female foxhounds, weighing 28 ± 1 kg) treated in accordance with NIH guidelines for animal care. Experiments were performed with approval of the local animal care and use committee (North Rhine-Westphalia State Agency for Nature, Environment and Consumer Protection, Recklinghausen, Germany; ref. 87-51.04.2010.A073).

Prior to the experiments, food was withheld overnight with water ad libitum to ensure complete gastric depletion and to avoid changes in perfusion and oxygenation due to digestive activity. Each dog underwent each experimental protocol in a randomized order and served as its own control. Experiments were performed at least 3 weeks apart to prevent carry-over effects. The experiments were performed under general anesthesia (induction of anesthesia with 4 mg·kg^−1^ propofol, maintenance with sevoflurane, end-tidal concentration 3.0%, and 1.5 MAC in dogs [21]). The animals were mechanically ventilated after endotracheal intubation (F_*i*_O_2_ = 0.3, VT = 12.5 mL·kg^−1^) with the respiratory frequency adjusted to achieve normocapnia (end-expiratory carbon dioxide, etCO_2_ = 35 mmHg), verified by continuous capnography (Capnomac Ultima, Datex Instrumentarium, Helsinki, Finland). During baseline conditions, the dogs were placed on their right side and covered with warming blankets to maintain body temperature at 37.5°C (continuous arterial measurement). Throughout the experiments, no additional fluid replacement was administered to avoid volume effects that could influence tissue perfusion and oxygenation. However, after withdrawal of each blood sample, normal saline was infused three times the sampling volume to maintain blood volume.

### 2.2. Measurements

#### 2.2.1. Systemic Hemodynamic and Oxygenation Variables

The aorta was catheterized via the left carotid artery for continuous measurement of mean arterial pressure (MAP, Gould-Statham pressure transducers P23ID, Elk Grove, IL) and intermittent arterial blood gas samples adjusted for temperature (Rapidlab 860, Bayer AG, Germany) from appropriate syringes (PICO 50, Radiometer, Copenhagen, Denmark). Oxygen saturation was calculated for canine blood from pO_2_ and adjusted to pH and temperature [22]. Arterial oxygen content (C_*a*_O_2_ = hemoglobin·1.34·oxygen saturation + pO_2_·0.0031) and DO_2_ (DO_2_ = C_*a*_O_2_·CO) were calculated subsequently. Cardiac output was determined via transpulmonary thermodilution (PiCCO 4.2 non US, PULSION Medical Systems, Munich, Germany) at the end of each intervention, at least every 30 minutes, as previously described [23, 24].

Heart rate (HR) was continuously measured by electrocardiography (Powerlab, ADInstruments, Castle Hill, Australia). All hemodynamic and respiratory variables were recorded on a personal computer after analog to digital conversion (Powerlab, ADInstruments, Castle Hill, Australia) for later analysis.

#### 2.2.2. Mucosal Oxygenation and Perfusion

*μ*HbO_2_ and *μ*flow of the gastric and oral mucosa were continuously assessed by tissue reflectance spectrophotometry and laser Doppler flowmetry (O2C, LEA Medizintechnik, Gießen, Germany), as detailed previously [24, 25]. 

Briefly, white light (450–1000 nm) and laser light (820 nm, 30 mW) are transmitted to the tissue of interest via a microlightguide and the reflected light is analyzed. The wavelength-dependent absorption and overall absorption of the applied white light can be used to calculate the percentage of oxygenated hemoglobin (*μ*HbO_2_) and the amount of hemoglobin (*μ*Hb) [26]. Due to the Doppler effect, magnitude and frequency distribution of changes in wavelength are proportional to the number of blood cells multiplied by the measured mean velocity (*μ*Vel) of these cells. This product is proportional to flow and expressed in arbitrary perfusion units (aU) [27]. Hence, this method allows assessment and comparison of oxygenation and perfusion of the same region at the same time. Changes of flow can be attributed either to change of velocity or number of red blood cells, comparable to the information gained by intravital microscopy. 

Since light is totally absorbed in vessels with a diameter >100 *μ*m [28] only microvascular oxygenation of nutritive vessels of the mucosa is measured. The biggest fraction of the blood volume is stored in venous vessels; therefore, mainly postcapillary oxygenation is measured which represents the critical partial pressure of oxygen (pO_2_) for ischemia [29]. 

One flexible lightguide probe is placed in the mouth facing the buccal side of the oral mucosa and a second probe is introduced into the stomach via an orogastric silicone tube and positioned facing the greater curvature [30]. Both sites of measurement represent the microcirculation of other gastrointestinal mucosa regions [31, 32]. Online evaluation of the signal quality throughout the experiments allows verification of the correct position of the probe tip. The *μ*HbO_2_ and *μ*flow values reported are the means of the last 5 min (150 spectra, 2 s each) of the respective intervention under steady state conditions. The nontraumatic instrumentation in particular non-traumatic access to the gastric mucosa, allows the determination of mucosal microcirculation in the absence of surgical stress. This is particularly desirable with respect to the marked alterations that surgical stress exerts on splanchnic circulation [33]. In this situation reflectance spectrophotometry reliably detects even clinically asymptomatic reductions in *μ*HbO_2_ [34] and highly correlates with the morphologic severity and extent of gastric mucosal tissue injury [35].

*μ*DO_2_ (*μ*DO_2_ = C_*a*_O_2_·*μ*flow) and postcapillary oxygen content (C_*k*_O_2_ = hemoglobin·1.34·*μ*HbO_2_) were calculated. Oral mucosal oxygen consumption (*μ*VO_2_) was calculated from the difference in arterial postcapillary oxygen content (ΔC_av_O_2_ = hemoglobin·1.34·systemic oxygen saturation − C_*k*_O_2_) and regional flow (*μ*VO_2_ = C_av_O_2_·regional flow). Since flow signals of the gastric probe did not have sufficient quality in some experiments, only flow signals of the oral mucosa are presented.

### 2.3. Induction of Hypothermia

Body temperature was reduced continuously over 90 minutes to achieve a core temperature of 34°C. Four commercially available cooling packs (Gello GmbH, Ahaus, Germany) were wrapped into towels and placed around the paws while crushed ice stored in bags was wrapped into towels and placed on the body. Body temperature was continuously measured via arterial catheter (PiCCO 4.2). When temperature fell below 34°C, forced-air warming (Bair Hugger, Model 500, Augustine Medical Inc.) at 38°C was used to maintain the body temperature at 34°C. At the end of the experiment normal body temperature for dogs (37–38.5°C) was restored using forced-air warming.

### 2.4. Induction of Hemorrhagic Shock

Hemorrhagic shock was induced by removing 20% of the estimated total blood volume via a large bore intravenous catheter in a peripheral vein and the arterial catheter (i.e., 16 mL·kg^−1^ of whole blood over five minutes). According to Advanced Trauma Life Support this model represents a class II shock (blood loss 15–30%) [36]. This reversible and nonlethal shock model allows the investigation of either protective or harmful effects of various interventions, that is, hypothermia. Heparinized shed blood was stored and later retransfused using an infusion set with a 200 *μ*m filter.

### 2.5. Experimental Protocol

**Figure 1:**
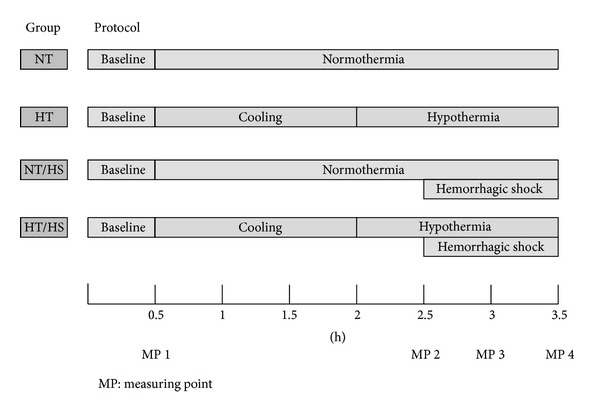
Experimental protocol: normothermia (NT), hypothermia (HT), normothermia during hemorrhagic shock (NT/HS), and hypothermia during hemorrhagic shock (HT/HS).

After instrumentation, 30 min was allowed to establish steady state conditions and baseline values were recorded before the animals were randomized to the respective protocol (Figure 1). Steady state conditions were defined as stability of hemodynamic variables (heart rate and mean arterial pressure) as well as ventilation parameters (end-tidal CO_2_, end-tidal sevoflurane concentration, and inspiratory oxygen fraction).

#### 2.5.1. Hypothermia (HT)

To study the effects of hypothermia on *μ*HbO_2_, body temperature was reduced over 90 minutes. Hypothermia was maintained for two hours and all variables were recorded. Normal body temperature was restored over 120 minutes.

#### 2.5.2. Control Experiment, Normothermia (NT)

As time control experiment, body temperature was kept at 37.5°C without any further intervention.

#### 2.5.3. Hypothermia + Hemorrhagic Shock (HT/HS)

To study the effect of hypothermia on hemorrhage, hypothermia was induced as described above. During hypothermia, hemorrhagic shock was induced and maintained for 60 minutes, followed by retransfusion of the shed blood and rewarming of the animal.

#### 2.5.4. Control Experiment, Normothermia + Hemorrhagic Shock (NT/HS)

The effects of hemorrhage alone on *μ*HbO_2_ were studied under normothermia. Hemorrhagic shock was induced 120 minutes after baseline recording, followed by retransfusion of the shed blood.

Blood samples were obtained for blood gas analysis at the indicated measuring points (Figure 1). These time points reflect a steady state under baseline conditions (MP 1), under hypothermia after reaching the intended temperature of 34°C (MP 2) and during hemorrhage (MP 3 + 4).

### 2.6. Statistical Analysis

Data for analysis were obtained during the last five minutes of each intervention under steady state conditions. All data are presented as mean ± standard error (mean ± SE) for *n* = 5 animals. Normal data distribution was assessed and confirmed in Q-Q plots (IBM SPSS Statistics, International Business Machine Corp., USA). Differences within the groups and between the groups were tested using a Wilcoxon signed-rank test (StatView V4.1, SAS Institute Inc, Cary, NC, USA); *P* < 0.05 was considered significant.

## 3. Results

**Figure 2:**
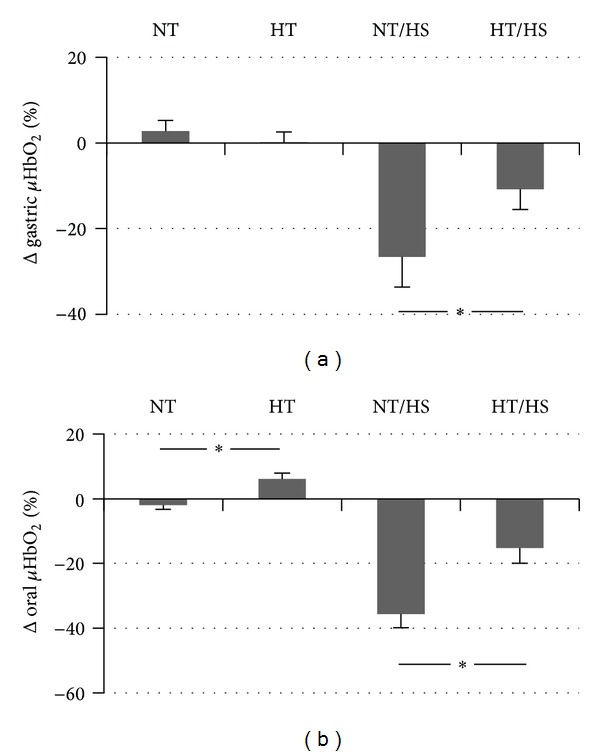
Effect of normothermia (NT), hypothermia (HT), hemorrhage during normothermia (NT/HS), and hemorrhage during hypothermia (HT/HS) on gastric and oral microvascular hemoglobin oxygen saturation (*μ*HbO_2_). Effects of hemorrhage: reduction of *μ*HbO_2_ after 30 minutes of shock (3.0 h) versus *μ*HbO_2_ before shock (2.5 h) during normothermia (NT/HS) and hypothermia (HT/HS). Effect of hypothermia without hemorrhage: change of *μ*HbO_2_ at the corresponding time point (3.0 h) versus baseline conditions under normothermia (NT) and hypothermia (HT). Data are presented as absolute changes for *n* = 5 dogs, mean ± SE, *P < 0.05.

**Figure 3:**
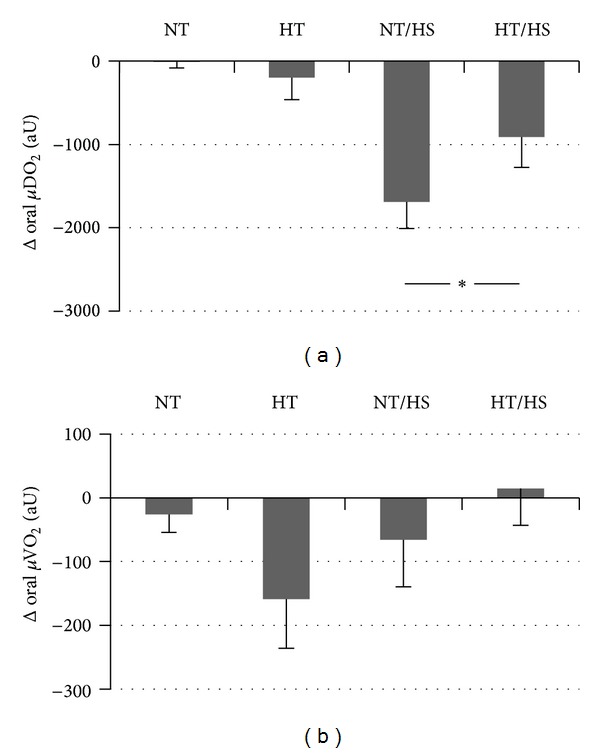
Effect of normothermia (NT), hypothermia (HT), hemorrhage during normothermia (NT/HS), and hemorrhage during hypothermia (HT/HS) on oral mucosal oxygen delivery (*μ*DO_2_) and oxygen consumption (*μ*VO_2_). Effects of hemorrhage: change of *μ*DO_2_ and VO_2_ after 30 minutes of shock (3.0 h) versus *μ*DO_2_ and *μ*VO_2_ before shock (2.5 h) during normothermia (NT/HS) and hypothermia (HT/HS). Effect of hypothermia without hemorrhage: change of *μ*DO_2_ and *μ*VO_2_ at the corresponding time point (3.0 h) versus baseline conditions under normothermia (NT) and hypothermia (HT). Data are presented as absolute changes for *n* = 5 dogs, mean ± SE, *P < 0.05.

**Figure 4:**
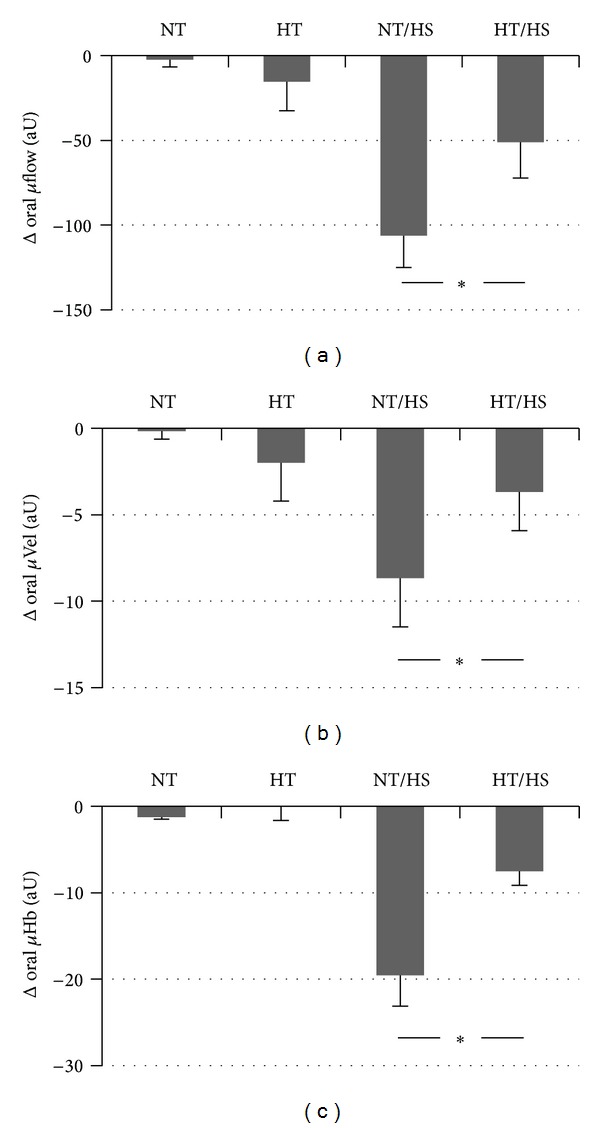
Effect of normothermia (NT), hypothermia (HT), hemorrhagic shock during normothermia (NT/HS), and hemorrhage during hypothermia (HT/HS) on oral mucosal perfusion (*μ*flow), red blood cell velocity (*μ*Vel), and amount of tissue haemoglobin (*μ*Hb). Effects of hemorrhage: change of *μ*flow, *μ*Vel, and *μ*Hb after 30 minutes of shock (3.0 h) versus *μ*flow, *μ*Vel, and *μ*Hb before shock (2.5 h) during normothermia (NT/HS) and hypothermia (HT/HS). Effect of hypothermia without hemorrhage: change of *μ*flow, *μ*Vel, and *μ*Hb at the corresponding time point (3.0 h) versus baseline conditions under normothermia (NT) and hypothermia (HT). Data are presented as absolute changes for *n* = 5 dogs, mean ± SE, *P < 0.05.

Hypothermia alone (HT) did not influence gastric *μ*HbO_2_ compared to baseline conditions (Figure 2). In contrast, hypothermia significantly increased oral *μ*HbO_2_ by +6 ± 2%. This was related to a reduction in *μ*VO_2_ of −159 ± 76 aU during hypothermia compared to almost unchanged *μ*VO_2_ (−26 ± 28 aU) during normothermia at the same time point, which, however, failed to reach significance (*P* = 0.08). Hypothermia neither changed DO_2_ (Table 1) nor *μ*DO_2_ (Figure 3). The increase of oral *μ*HbO_2_ was independent of *μ*flow which did neither change during normothermia (−2 ± 4 aU) nor during hypothermia (−16 ± 17 aU). Accordingly, *μ*Vel and *μ*Hb remained unchanged as well (Figure 4).

### 3.1. Hypothermia + Hemorrhage

During control experiments (37.5°C) with hemorrhage alone (NT/HS), gastric *μ*HbO_2_ decreased by −27 ± 7% and oral *μ*HbO_2_ by −36 ± 4% after 30 minutes of hemorrhage (Figure 2). This decrease was significantly attenuated during hypothermia for both oral and gastric *μ*HbO_2_ (gastric *μ*HbO_2_ decreased by −11 ± 4 and oral *μ*HbO_2_ by −15 ± 5%) (Figure 2). This effect was not related to DO_2_ which was reduced during hemorrhagic shock independent of normo- or hypothermic conditions almost equally by −5 ± 1 mL/kg/min (normothermia) and by −5 ± 1 mL/kg/min (hypothermia). Reduction of DO_2_ was caused by a lower CO that was equally reduced in both groups without any differences between normothermia and hypothermia.

The differences of *μ*HbO_2_ during hemorrhage are related to a decrease of *μ*DO_2_ during normothermia (−1690 ± 319 aU) that was ameliorated during additional hypothermia (−910 ± 366 aU), while *μ*VO_2_ did not change in both groups (Figure 3). These differences in *μ*DO_2_ are based on changes of *μ*flow that decreased during hemorrhage under normothermia (−106 ± 19 aU) and improved under hypothermia (−51 ± 21 aU). Higher *μ*flow values are attributed to both higher *μ*Vel and higher *μ*Hb (Figure 4). *μ*Vel decreased during hemorrhage by −9 ± 3 aU under normothermia but only by −4 ± 2 aU under hypothermia. In addition, hypothermia attenuated the decrease of *μ*Hb from −20 ± 4 during normothermia to −8 ± 2 aU.

## 4. Discussion

The aim of our study was to evaluate the effects of hypothermia on oral and gastric *μ*HbO_2_ and *μ*flow under physiological and hemorrhagic conditions. We have observed in this study that hypothermia increases oral *μ*HbO_2_ under physiological conditions without influencing gastric *μ*HbO_2_. According to our results, hypothermia attenuates the effects of hemorrhage on oral and gastric *μ*HbO_2_. This effect could be related to the observed increase of *μ*DO_2_ based on the increase of *μ*flow with unchanged *μ*VO_2_.

These results are quite interesting and of clinical importance, since hypothermia is widely used after resuscitation, but data on the effect of hypothermia on oral and gastric *μ*HbO_2_ are lacking so far. Since hypothermia did not affect gastric *μ*HbO_2_ under otherwise physiological conditions, its benefit as a therapeutic approach to improve gastric oxygenation remains controversial. Our data did not reveal any detrimental effects and hypothermia might thus be applied after resuscitation without further compromising gastric microcirculation. Nevertheless, other variables and thus possible negative effects of hypothermia have to be considered as well. Additionally, the presented data are derived from an animal study on five dogs. Therefore, their impact on the therapeutic application in humans has to be interpreted with care.

Additionally, both groups investigated during normothermia (NT and NT/HS) and accordingly both groups investigated during hypothermia (HT and HT/HS) were treated equally before hemorrhage (MP 2). Those groups were not tested for significant differences; however, suspected differences might be attributed to minor differences in baseline values. To exclude differences in baseline values as confounding factor, our main findings are based on relative changes of the variables (Figures 2–4) and not on absolute values.

Oral *μ*HbO_2_ increased under hypothermia whereas gastric *μ*HbO_2_ remained unchanged. Oral microcirculation, like gastric microcirculation, is known to represent microcirculation of other gastrointestinal mucosa regions [31, 32]. Our observed results indicate that *μ*HbO_2_ is improved in some regions of the gastrointestinal tract while others remain unchanged. This increase is observed without changes in blood flow or *μ*DO_2_ and might be related to reduced metabolism and thus reduced *μ*VO_2_ under hypothermia. It remains speculative why hypothermia increased oral but not gastric *μ*HbO_2_ during physiological conditions. One explanation could be the difference in local temperature which might be lower in the oral mucosa as a more peripheral compartment compared to the gastric mucosa as a more central organ. Though oral temperature was not measured, this local hypothermia could induce a higher reduction of metabolism and *μ*VO_2_ in the oral compared to the gastric mucosa.

Concerning the effect of hypothermia during hemorrhage we could show in the present study that oral and gastric mucosa are partially protected by hypothermia during hemorrhage. Interestingly, the higher level of *μ*HbO_2_ during hemorrhage under hypothermia is associated with a higher *μ*DO_2_ and an unchanged DO_2_. Similarly, *μ*flow is increased without increase of CO. Thus, the observed effects might be related to local microcirculatory vasoregulation rather than alterations of systemic circulation. There are several possible reasons for the observed increase of *μ*HbO_2_ and *μ*flow. *μ*HbO_2_ increases due to increased oxygen supply, that is, DO_2_, or due to a reduction of *μ*VO_2_. The reduced oxygen consumption could be related to a reduced oxygen demand or to the inability to extract oxygen. The inability to extract oxygen might be related to the left shift of the oxygen-hemoglobin dissociation curve during hypothermia with an increased oxygen binding capacity of hemoglobin. The measurement of *μ*flow in our experiments demonstrates that the observed increase of *μ*HbO_2_ seems to be linked to an increased *μ*flow and *μ*DO_2_ rather than changes of *μ*VO_2_. Thus, the improved oxygenation during hemorrhage in this study seems not to be based on reduced oxygen consumption or to the inability to extract oxygen. The increase of *μ*flow might be attributed to higher *μ*Vel and higher *μ*Hb (Table 2). Increase of *μ*Hb is particularly desirable whereas high velocity solely can occur in regions with low capillary density and extended diffusion distances. In contrast, *μ*Hb correlates with the amount of blood in the tissues and thus probably indicates high capillary density. 

Being concordant with our results others showed an increase in portal venous flow during hemorrhage [16] while data on microcirculatory oxygenation are lacking so far. In contrast to our results, red blood cell flux of the gastric mucosa was reduced during hypothermia in humans [37]. However, this study was conducted during cardiopulmonary bypass with several confounders, for example, extracorporeal circulation, insertion depths of the venous cannulas, or the release of proinflammatory cytokines. Nevertheless, during hemorrhage survival was increased under hypothermia after 24 h in a hemorrhagic shock model in rats [19]. Though bacterial translocation was not studied, other data indicates that regional hypothermia during gut ischemia protects against histological injury and impaired intestinal transit [38]. The reasons for these effects are still unknown but could be attributed to a reduced ischemia/reperfusion injury [39], to induction of heme oxygenase-1 [38], or, according to our results, to an increased splanchnic oxygenation and perfusion. 

Besides the potential protective effects of hypothermia negative effects have to be taken into account. It has been shown that hypothermia reduces blood clotting during hemorrhage and reduced survival in trauma patients [40]. Furthermore, hypothermia triggers shivering with a considerable increase of oxygen consumption and patients therefore require deep sedation. Additional negative effects can be ameliorated by a relatively slow induction phase and a fast rewarming [14]. For these reasons its use will stay restricted to certain conditions, like postcardiac arrest and during heart surgery.

Measurement of flow or *μ*HbO_2_ alone cannot serve as an indicator of oxygen supply per se. The assessment of flow does not distinguish between impaired and increased oxygen supply and the measurement of *μ*HbO_2_ does not distinguish between increased oxygen supply and the inability to extract oxygen. Our approach to analyze oxygenation and perfusion and thus *μ*DO_2_ as well as *μ*VO_2_ seems to be more reliable than the singularly approach of other studies analyzing either perfusion or oxygenation. Additionally, splanchnic oxygenation and perfusion are not necessarily linked and thus have to be evaluated separately. Another advantage is our assessment of splanchnic oxygenation at two independent measurement sites. The assessment of *μ*Vel and *μ*Hb allows evaluation of the reasons for changes in *μ*flow and gives information comparable to intravital microscopy with additional analysis of oxygenation that cannot be assessed by microscopy.

*Limitations of This Study.* This is an animal study with *n* = 5 dogs. The number of animals seems to be rather small since we are forced by law to minimize animal experiments. However, the use of a cross-over design where each animal serves as its own control and eliminates interindividual differences warrants the use of a rather small number of animals. However, we did not correct for multiple comparisons. This increases the power to detect differences with small sample sizes. Correcting for multiple comparisons requires a large amount of dogs or reduces the power to detect effects (increasing type II error). This, in contrast, includes an increased chance for type I errors. Thus, the implications of our results have to be interpreted with care and the results of this animal study have to be analysed in a clinical setting in the future.

## 5. Conclusions

In this study, hypothermia improves oral and gastric *μ*HbO_2_ and oral *μ*flow during hemorrhagic shock in dogs. This effect might be explained by an increase of *μ*DO_2_ on the basis of increased *μ*flow with unchanged *μ*VO_2_.

## Figures and Tables

**Table 1:** Hemodynamic variables of the experimental groups.

Variable	Group	Measuring point 1 (0.5 h)	Measuring point 2 (2.5 h)	Measuring point 3 (3.0 h)	Measuring point 4 (3.5 h)
Gastric *μ*HbO_2_ [%]	NT	70 ± 4	70 ± 4	68 ± 5	67 ± 5
	HT	72 ± 2	73 ± 2	73 ± 2	73 ± 1
	NT/HS	76 ± 1	78 ± 2	51 ± 7*	56 ± 8*
	HT/HS	74 ± 1	71 ± 2	60 ± 6^#^	60 ± 8

Oral *μ*HbO_2_ [%]	NT	79 ± 2	82 ± 1	81 ± 1	80 ± 1
	HT	80 ± 2	85 ± 3*	86 ± 1^∗#^	84 ± 1
	NT/HS	75 ± 2	78 ± 1	42 ± 5*	48 ± 4*
	HT/HS	76 ± 1	81 ± 1^#^	65 ± 4^#^	62 ± 4*

*μ*flow [aU]	NT	127 ± 29	129 ± 24	125 ± 26	119 ± 25
	HT	140 ± 33	124 ± 26	125 ± 22	122 ± 21
	NT/HS	146 ± 30	160 ± 25	53 ± 10*	63 ± 13*
	HT/HS	131 ± 22	136 ± 20	85 ± 18*	78 ± 16

*μ*Vel [aU]	NT	23 ± 3	23 ± 3	23 ± 3	22 ± 3
	HT	27 ± 4	25 ± 2	25 ± 2	26 ± 2
	NT/HS	25 ± 3	26 ± 3	17 ± 3	18 ± 3*
	HT/HS	26 ± 3	28 ± 4	25 ± 4	25 ± 4

*μ*Hb [aU]	NT	87 ± 3	86 ± 3*	85 ± 3*	85 ± 3*
	HT	84 ± 2	84 ± 3	84 ± 2	84 ± 2
	NT/HS	87 ± 3	84 ± 4	64 ± 7*	67 ± 7*
	HT/HS	83 ± 3	81 ± 3	73 ± 4*	73 ± 3*

*μ*DO_2_ [aU]	NT	2082 ± 481	2123 ± 411	2073 ± 440	1976 ± 430
	HT	2374 ± 558	2195 ± 433	2177 ± 382	2135 ± 368
	NT/HS	2345 ± 481	2517 ± 411	828 ± 154*	982 ± 187*
	HT/HS	2187 ± 359	2327 ± 345	1417 ± 262*	1310 ± 262

DO_2_ [mL·kg^−1^·min^−1^]	NT	15 ± 1	14 ± 1	14 ± 1	14 ± 1
	HT	14 ± 1	13 ± 1	13 ± 1	13 ± 1
	NT/HS	14 ± 1	13 ± 1	8 ± 1*	9 ± 1*
	HT/HS	15 ± 1	14 ± 1	9 ± 1^∗#^	10 ± 1*

VO_2_ [aU]	NT	380 ± 64	351 ± 62	354 ± 62	349 ± 64
	HT	423 ± 92	273 ± 27	265 ± 23*	318 ± 53
	NT/HS	527 ± 108	512 ± 90	447 ± 85	474 ± 88
	HT/HS	472 ± 72	421 ± 59	435 ± 67	453 ± 85

SVR [mmHg·L^−1^·min]	NT	26 ± 2	27 ± 2	28 ± 2*	28 ± 2*
	HT	27 ± 2	28 ± 2	29 ± 2	30 ± 2*
	NT/HS	26 ± 2	26 ± 2	35 ± 2	35 ± 2*
	HT/HS	25 ± 2	27 ± 2	33 ± 2	34 ± 2

CO [mL·kg^−1^·min^−1^]	NT	88 ± 8	85 ± 6	84 ± 5	84 ± 5
	HT	81 ± 7	74 ± 4	73 ± 3	72 ± 4^∗#^
	NT/HS	88 ± 6	85 ± 3	52 ± 3*	59 ± 3*
	HT/HS	87 ± 6	80 ± 3	53 ± 3*	57 ± 4*

SV [mL]	NT	23 ± 2	22 ± 2	22 ± 2*	22 ± 2*
	HT	22 ± 2	27 ± 2^∗#^	26 ± 2^∗#^	26 ± 2^∗#^
	NT/HS	22 ± 2	22 ± 2	14 ± 1*	15 ± 1*
	HT/HS	23 ± 2	27 ± 3^∗#^	16 ± 1^∗#^	17 ± 1^∗#^

MAP [mmHg]	NT	63 ± 2	65 ± 1	65 ± 1	65 ± 2
	HT	62 ± 2	61 ± 1^#^	61 ± 1	61 ± 2
	NT/HS	65 ± 1	65 ± 1	53 ± 1*	61 ± 2
	HT/HS	62 ± 2	62 ± 3	50 ± 4*	55 ± 3^∗#^

HR [min^−1^]	NT	111 ± 6	111 ± 5	111 ± 5	110 ± 6
	HT	108 ± 5	82 ± 4^∗#^	81 ± 4^∗#^	81 ± 4^∗#^
	NT/HS	117 ± 5	112 ± 4*	114 ± 8	117 ± 8
	HT/HS	112 ± 6^#^	88 ± 6^∗#^	96 ± 8^∗#^	97 ± 8^∗#^

Effect of normothermia (NT), hypothermia (HT), hemorrhage during normothermia (NT/HS), and hemorrhage during hypothermia (HT/HS) on gastric and oral mucosal hemoglobin oxygenation (*μ*HbO_2_), microvascular flow (*μ*flow), velocity (*μ*Vel) and amount of haemoglobin (*μ*Hb), regional (*μ*DO_2_), and systemic oxygen delivery (DO_2_), oral mucosal oxygen consumption (*μ*VO_2_), systemic vascular resistance (SVR), cardiac output (CO), stroke volume (SV), mean arterial pressure (MAP), and heart rate (HR); data are presented as absolute values, mean ± SE, *n* = 5, *P < 0.05 versus baseline, ^#^ *P* < 0.05 versus NT for group HT and versus NT/HV for group HT/HV.

**Table 2:** Metabolic and respiratory variables of the experimental groups.

Variable	Group	Measuring point 1 (0.5 h)	Measuring point 2 (2.5 h)	Measuring point 3 (3.0 h)	Measuring point 4 (3.5 h)
S_a_O_2_ [%]	NT	99 ± 1	99 ± 1	99 ± 1	99 ± 1
	HT	99 ± 1	99 ± 1^∗#^	99 ± 1*	99 ± 1^∗#^
	NT/HS	99 ± 1	99 ± 1	98 ± 1*	98 ± 1*
	HT/HS	99 ± 1	99 ± 1^∗#^	98 ± 1^#^	98 ± 1^∗#^

pCO_2_ [mmHg]	NT	39 ± 1	39 ± 1	40 ± 1	39 ± 1
	HT	40 ± 1	41 ± 1*	40 ± 1	41 ± 1^∗#^
	NT/HS	40 ± 1	42 ± 1	45 ± 1*	44 ± 1*
	HT/HS	39 ± 1	40 ± 1*	44 ± 1*	43 ± 1*

pO_2_ [mmHg]	NT	160 ± 4	164 ± 1	161 ± 2	164 ± 2
	HT	157 ± 1	159 ± 2^#^	156 ± 3	160 ± 3
	NT/HS	155 ± 2	158 ± 2	147 ± 3*	151 ± 2
	HT/HS	158 ± 3	163 ± 3	148 ± 2*	149 ± 3*

PH	NT	7,42 ± 0,01	7,40 ± 0,01*	7,39 ± 0,01*	7,39 ± 0,01*
	HT	7,41 ± 0,01	7,39 ± 0,01*	7,39 ± 0,01	7,38 ± 0,01*
	NT/HS	7,39 ± 0,01	7,38 ± 0,01	7,33 ± 0,01*	7,34 ± 0,01*
	HT/HS	7,41 ± 0,01	7,38 ± 0,01*	7,33 ± 0,01*	7,33 ± 0,01*

Hb [g·100 mL^−1^]	NT	12 ± 1	12 ± 1	12 ± 1	12 ± 1
	HT	13 ± 1	13 ± 1^#^	13 ± 1*	13 ± 1^#^
	NT/HS	12 ± 1	12 ± 1	12 ± 1	12 ± 1
	HT/HS	12 ± 1	13 ± 1	12 ± 1	12 ± 1

Lactate [mmol·L^−1^]	NT	0,6 ± 0,1	0,9 ± 0,1	1,0 ± 0,1	0,9 ± 0,1*
	HT	1,3 ± 0,2^#^	1,1 ± 0,2^∗#^	1,1 ± 0,2^∗#^	1,1 ± 0,2^∗#^
	NT/HS	0,9 ± 0,3	1,2 ± 0,2	1,3 ± 0,2	1,2 ± 0,2
	HT/HS	0,8 ± 0,1	0,7 ± 0,1	0,7 ± 0,1^#^	0,7 ± 0,1^#^

Effect of normothermia (NT), hypothermia (HT), hemorrhage during normothermia (NT/HS), and hemorrhage during hypothermia (HT/HS) on systemic oxygen saturation (SAT), carbon dioxide partial pressure (pCO_2_), oxygen partial pressure (pO_2_), pH, hemoglobin (Hb), and lactate; data are presented as absolute values, mean ± SE, *n* = 5, *P < 0.05 versus baseline, ^#^ *P* < 0.05 versus NT for group HT and versus NT/HV for group HT/HV.
